# Mapping temporal-network percolation to weighted, static event graphs

**DOI:** 10.1038/s41598-018-29577-2

**Published:** 2018-08-17

**Authors:** Mikko Kivelä, Jordan Cambe, Jari Saramäki, Márton Karsai

**Affiliations:** 1 Department of Computer Science, Aalto University School of Science, FI-00076 Aalto, Finland; 2 Univ Lyon, ENS de Lyon, Inria, CNRS, UCB Lyon 1, LIP UMR 5668, IXXI, F-69342 Lyon, France

## Abstract

The dynamics of diffusion-like processes on temporal networks are influenced by correlations in the times of contacts. This influence is particularly strong for processes where the spreading agent has a limited lifetime at nodes: disease spreading (recovery time), diffusion of rumors (lifetime of information), and passenger routing (maximum acceptable time between transfers). We introduce weighted event graphs as a powerful and fast framework for studying connectivity determined by time-respecting paths where the allowed waiting times between contacts have an upper limit. We study percolation on the weighted event graphs and in the underlying temporal networks, with simulated and real-world networks. We show that this type of temporal-network percolation is analogous to directed percolation, and that it can be characterized by multiple order parameters.

## Introduction

Contact network structure plays an important role in many dynamical processes, in particular in diffusion-like phenomena^1,2^. Recently, the temporal properties of networks have been shown to strongly influence spreading dynamics^3–5^. This is (a) because spreading processes must follow causal, time-respecting paths spanned by sequences of contacts^6–10^ and (b) because the speed and ability of spreading processes to percolate through the contact structure are affected by temporal inhomogeneities such as the burstiness of contacts, visible as broad inter-contact time distributions^11–15^ and correlated contact times^16–18^. However, this picture is still lacking detail, especially when it comes to percolation.

Processes with limited waiting times at nodes are particularly sensitive to broad distributions of inter-contact times; the longest inter-contact times may stop the process. Such processes include variants of epidemiological models such as Susceptible-Infectious-Recovered (SIR) and Susceptible-Infectious-Susceptible (SIS)^11,19–27^ where nodes only remain infectious for finite periods. Other examples include social contagion^28,29^ ad-hoc message passing by mobile agents^30^ and passenger routing^31^.

In these processes, the spreading agent must be transmitted onward from a node within some time *δt* or the process stops. One can imagine a timer that starts ticking whenever the infection/message arrives at a node: this infection/message can only be transmitted onward through those of the node’s contacts that happen before the timer reaches *δt*. This waiting time limit can be directly incorporated into time-respecting paths by requiring that their successive contacts are separated by no more than *δt* units of time. Because these are the only paths that the spreading process can follow, its outcome then depends on the existence of such paths. For very low values of *δt*, network-wide connectivity is unlikely and spreading processes do not percolate the network, whereas a large value *δt* may provide the pathways for infecting most of the network.

Therefore, the time limit *δt* is the control parameter of a percolation problem, where connectivity is determined by paths of contacts that follow one another within *δt*. However, discovering all paths separately for each value of *δt* is computationally expensive; a faster way would be to compute paths for a range of values, taking use of redundancy. In this article, we introduce the weighted event graph as solution to the computational problem of temporal-network percolation, and use this representation to study percolation in artificial and real networks. We show that in temporal-network percolation, there are three types of order parameters, measured in terms of component nodes, events, and lifetime and that temporal-network percolation has strong connections to directed percolation.

Weighted event graphs are static, weighted, and directed acyclic graphs (DAGs) that encapsulate the complete set of *δt*-constrained time-respecting paths for all values of *δt* simultaneously. The subset of paths corresponding to a specific value of *δt* can be quickly extracted from the weighted event graph by simply thresholding it. Weighted event graphs can be viewed as a temporal-network extension of the line-graph representation of static networks. There is some similarity with the approach of ref.^10^ that maps two-event sequences onto aggregated second-order networks, and with that of ref.^32^ where an unweighted event graph is constructed from pairs of temporally closest events. Our approach builds on concepts introduced in refs^17,33^.

## Results

**Figure 1:**
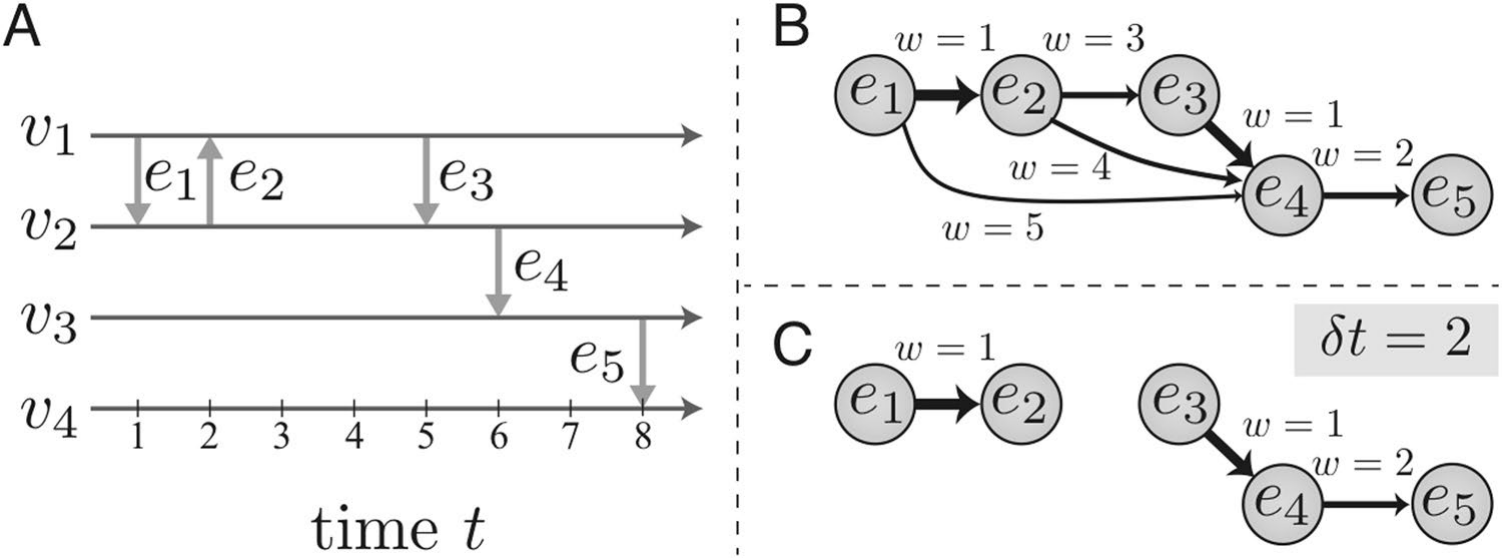
Constructing and thresholding the weighted event graph. (**a**) The time line of a temporal network with four nodes *v*_1_ − *v*_4_ and five events *e*_1_ − *e*_5_. (**b**) The weighted event graph representation of the temporal network. (**c**) The thresholded event graph, containing only pairs of events with a maximum time difference of *δt* = 2.

Let us consider a temporal network *G* = (*V*, *E*, *T*) with edges defined as a set of events $E\subset V\times V\times [0,T]$ over a time period *T* (e.g. see Fig. 1a). No self-edges or simultaneous events of the same node are allowed. Two events *e* = (*u*, *v*, *t*) and $e^{\prime} =(u^{\prime} ,v^{\prime} ,t^{\prime} )$ are considered adjacent so that $e\to e^{\prime}$ if they share at least one node and $t < t^{\prime}$. This definition of adjacency is directed and preserves the arrow of time. Further, two adjacent events are considered *δt*-adjacent if their temporal distance, i.e., their time difference, is $0 < t^{\prime} -t < \delta t$. The weighted event graph representation of *G* is defined as the graph *D* = (*E*, *E*_*D*_, *w*) where the set of nodes *E* is the set of events in *G* and the edges *e*_*D*_ ∈ *E*_*D*_ represent the adjacency of the events ${e}_{D}=e\to e^{\prime}$ with weights defined as temporal distances $w({e}_{D})=t^{\prime} -t$ (see Fig. 1b). That is, *D* is a directed acyclic graph with links weighted with temporal distances, contains all time-respecting paths in *G*. For paths with a waiting time limit *δt*, we get the subgraph *D*_*δt*_ by thresholding *D* so that only links with $w\le \delta t$ are retained (see Fig. 1c).

The *δt*-thresholded event graph *D*_*δt*_ is a superposition of the time-respecting paths that a *δt*-limited spreading process can follow. Therefore, its structure tells if the process can percolate the network. A closer look at the problem reveals that here, the concept of percolation is more complex than for static networks. The components of *D*_*δt*_ are directed, (even if the events of *G* are undirected). There are only weakly connected components–there are no strongly connected components because *D*_*δt*_ is by definition acyclic. Each event graph node has an in-component and out-component that contain events on up- and downstream temporal paths; these components may overlap for different event graph nodes^34^. In the following, we will limit our analysis to weakly connected components because of their uniqueness in *D*_*δt*_. For demonstration we computed the evolution of the largest in- and out-components as the function of *δt* for one empirical network as discussed in the Supplementary Materials (SM). For a spreading process to percolate, the existence of a weakly connected component is necessary but not sufficient.

The weighted event-graph representation allows us to employ computational tools developed for static networks to track percolation on temporal networks. We can sweep through the whole range of *δt* by starting from an empty graph and constructing each *D*_*δt*_ by adding links in the increasing order of their weight *w*(*e*_*D*_), and at the same time keeping track of the weakly-connected components after each addition (for further discussion see Materials and Methods). The computational cost of such process is dominated by the sorting of the edges in *D*. Their number scales in the worst case as ${\mathscr{O}}(|E|{s}_{{\rm{\max }}})$, where ${s}_{{\rm{\max }}}$ is the maximum number of events containing the same node in the temporal network (for details on computational complexity, see Materials and Methods). One could also use conventional algorithms for temporal networks^35^ for example to calculate the exact maximum out-component size for each value of *δt* by starting a breadth-first search for each node before each of its events and for each possible value of *δt* separately. This would, however, be extremely slow with worst-case scaling of ${\mathscr{O}}(|E{|}^{3})$. The dramatical increase in calculation speed that weighted event graphs provide comes at a cost: the weakly connected component size only gives an upper bound for the maximum out-component (and in-component) size and the memory requirements increase as one needs to store the weighted event graph *D*_*δt*_.

In percolation analysis, the relative size of the largest connected component is defined as the order parameter. Here, there are three ways of measuring the size of a component of *D*_*δt*_. (1) One can count the number of *event graph nodes* ${S}_{E}(E^{\prime} )=|E^{\prime} |$ in a connected component $E^{\prime} \subseteq E$ of *D*_*δt*_. This gives an upper bound for the number of events on the time-respecting paths that a spreading process can follow if it includes an event from that component. (2) One can count the number of *temporal*-*network nodes* ${S}_{G}(E^{\prime} )=|\,{\cup }_{(u,v,t)\in E^{\prime} }(u\cup v)|$ that are covered by the event graph component *E*′. This is an upper bound for the number of temporal-network nodes that any spreading process can reach via the component’s time-respecting paths. Note that a temporal-network node can belong to multiple event-graph components; this can result in multiple giant components that cover most nodes but are separated in time. (3) One can measure the *lifetime* of the event graph component ${S}_{LT}(E^{\prime} )=({{\rm{\max }}}_{(u,v,t)\in E^{\prime} }t-{{\rm{\min }}}_{(u,v,t)\in E^{\prime} }t)$. This is an upper bound for the lifetime of any spreading process on the component. Note that there can be many co-existing components with long (or infinite) lifetimes; frequent and sustained contacts between a small number of nodes can already induce such components.

With these measures, we can define the order parameter as the relative size of the largest connected component,

1 $${\rho }_{\ast }({D}_{\delta t})=\frac{1}{{N}_{\ast }}\mathop{{\rm{\max }}}\limits_{{n}_{S\ast }\ne 0}{S}_{\ast },$$

where ${n}_{{S}_{\ast }}$ is the number of components of size ${S}_{\ast }$ for the chosen definition of size * $\in \{E,G,LT\}$, and N_*_ is the maximum possible value that S_*_ can get as a single component, i.e., ${N}_{E}=|E|$, ${N}_{G}=|V|$, and *N*_*LT*_ = *T*. In conventional percolation analysis, the average size of the other connected components is a quantity of interest that is equivalent to magnetic susceptibility. It can be introduced for the ${S}_{\ast }(E^{\prime} )$ event graph components in *D*_*δt*_ as

2 $${\chi }_{\ast }({D}_{\delta t})=\frac{1}{{N}_{\ast }}\sum _{{S}_{\ast } < \,{\rm{\max }}\,{S}_{\ast }}{n}_{{S}_{\ast }}{S}_{\ast }^{2}.$$

One would expect this quantity to have a maximum at the critical *δt*_*c*_, where the percolating connected component emerges in the event graph; in the thermodynamic limit this maximum would become a singularity. However, this quantity might behave differently for *S*_*G*_(*E*′) and *S*_*LT*_(*E*′) components due to $\sum {n}_{{S}_{\ast }}$ not being a conserved quantity, and because of the possible multiplicity of giant components in these representations.

Note the link to *directed percolation*^36^, where there are two correlation lengths, temporal and spatial, characterizing correlations parallel and perpendicular to the directed lattice. In our case, the arrow of time defines the direction. However, instead of the regular lattice typical for directed percolation, our process unfolds on an irregular structure determined by the set of events that take place at each point in time. In this setting *ρ*_*E*_ gives the probability that a randomly selected event in *D*_*δt*_ belongs to a structurally percolating infinite cluster, while *ρ*_*LT*_ is the typical temporal correlation length for a given *δt*. In our case these correspond to two different order parameters, as the largest and most long-lived components might not be the same, unlike for directed percolation. Note that although we operate with the weakly connected component of *D*_*δt*_ for computational reasons, it is still embedded in time, and therefore it conserves the strongly anisotropic nature of the percolation process. The weakly connected component provides an upper estimate of the size or duration of the largest out-component, that is, an upper bound in terms of any of the order parameters.

### Weighted event graphs of modelled temporal networks

To explore how *δt* controls temporal-network connectivity, we introduce a simple toy model. We define an ensemble of temporal networks ${{\mathscr{G}}}_{p,r}(n,k,\alpha )$ where the topology is that of an ErdŐs-Rényi (E-R) random graph with *n* nodes and average degree *k*, and events are generated on each link by a Poisson process with *α* events per link on average. We set the observation period *T* long enough so that $\delta t\ll T$ and $\alpha \ll T$.

In this model, there is a transition from the disconnected to the connected phase when the independent Poissonian events become *δt*-adjacent and form a giant weakly connected component in *D*_*δt*_. In terms of degree, a lower bound for this critical point can be estimated as the point where the average out-degree of the event graph becomes $\langle {k}_{{D}_{\delta t}}^{{\rm{out}}}\rangle =1$. In the underlying E-R network, each edge is adjacent to 2(*k* − 1) + 1 edges (including the edge itself), and therefore the average out-degree of *D*_*δt*_ is $\langle {k}_{{D}_{\delta t}}^{out}\rangle =\alpha \delta t[2(k-1)+1]$. The condition for the critical point can then be written as

3 $${k}_{c}=\frac{{(\alpha \delta t)}^{-1}-1}{2}+1\,{\rm{and}}\,\delta {t}_{c}=\frac{1}{\alpha \mathrm{(2}k-\mathrm{1)}}.$$

**Figure 2:**
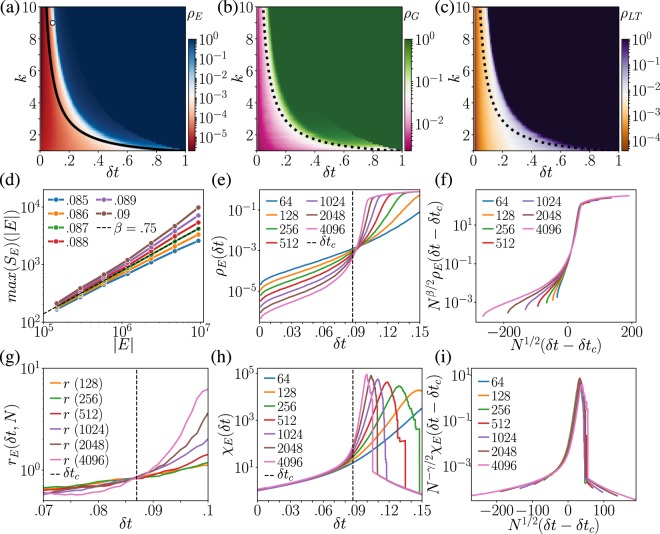
Phase diagrams for the random temporal network model as a function of the average network degree *k* and the maximum waiting time between events, *δt*. The color maps show the (ensemble-averaged) relative size ${\rho }_{\ast }(k,\delta t)$ of the giant weakly connected components, measured as (**a**) the number of events in the event graph components *S*_*E*_, (**b**) the number of temporal-network nodes that the largest event graph component covers, and (**c**) the lifetime of the event graph component *S*_*LT*_. The solid line in (**a**) (dashed lines in (**b**) and (**c**)) is the analytic estimate of Eq. (3). The circle in the upper left corner shows the critical point for *k* = 9 determined as explained in the text. (**d**) Scaling of ${\rm{\max }}({S}_{E})$, the size of the largest weakly connected component in *D*_*δt*_, with the size of *D*_*δt*_ measured in number of event-nodes |*D*_*δt*_| = |*E*|, for different *δt*. The dashed line assigns the critical *δt*_*c*_ = 0.87. (**e**) The order parameter *ρ*_*E*_(*δt*) for different network sizes *N* = |*V*| with *δt*_*c*_ shown as a dashed line. (**f**) Same as (**e**) after finite-size scaling using the function defined in Eq. (4). (**g**) The ratios *r*(*δt*, *N*) crossing at *δt*_*c*_. The dashed line shows the critical point determined in (**d**). (**h**) Susceptibility curves *χ*_*E*_(*δt*) for different sizes with *δt*_*c*_ shown as a dashed line. (**i**) Same as (**h**) after finite-size scaling using the function defined in Eq. (5). Computations for (**a**–**c**) are for a model network of |*V*| = 2048 nodes evolving for *T* = 512 time units with an event rate of *α* = 1 averaged over 10 realizations. Results for (**d**–**i**) have the same parameters but are averaged over 100 realizations and may differ in size.

This theoretical line *δt*_*c*_(*k*) is shown together with simulated results in Fig. 2a, with the number of events determining the relative size of the largest component, *ρ*_*E*_. *δt*_*c*_(*k*) separates the simulated percolating and non-percolating regimes well. Figure 2b and c show the relative largest component sizes in terms of temporal-network nodes (*ρ*_*G*_) and component lifetime (*ρ*_*LT*_); a percolation transition appears to take place near the theoretical line *δt*_*c*_(*k*) for the number of events from Eq. (3). Note generally, the phase transition lines for events, nodes, and lifetime can be different.

Let us investigate the model’s critical behavior in detail, fixing the average degree to *k* = 9. This makes the thresholded event graph *D*_*δt*_ dense enough for the mean-field (MF) approach; the MF approximation works well for regular lattices above the critical dimension *d*_*c*_ = 5. We locate the critical point with two methods. First, when the system reaches a stationary state where the order parameter becomes time-invariant beyond fluctuations, the scaling relation $max({S}_{E})\sim |{D}_{\delta t}{|}^{\beta }$ is expected to hold around the critical point *δt*_*c*_, where |*D*_*δt*_| is the size of the thresholded event graph in events, and *β* is the critical exponent of the order parameter. We measured this relation for several system sizes and values of *δt* and found a power-law scaling of *S*_*E*_(|*D*_*δt*_|) around $\delta {t}_{c}\simeq 0.087$ with the exponent $\beta \simeq 0.75$ (see Fig. 2d). This point is shown as a circle in Fig. 2a; it is above the analytical estimate, which provides the lower bound for the critical point. Note that for the directed-percolation university class, the MF solution suggests *β*_*MF*_ = 1.

The second way of determining the critical point is to calculate the ratios $r(\delta t,N)={\rho }_{E}(\delta t,N)/{\rho }_{E}(\delta t,N\mathrm{/2})$ for varying *N*^37^. These curves should cross around the critical point *δt*_*c*_ where $r(\delta {t}_{c},N)={2}^{-x}$, and *x* is related to the finite-size scaling exponent. In Fig. 2g, they indeed cross close to $\delta {t}_{c}\simeq 0.087$ with $r(\delta t)\simeq 0.82$ suggesting an exponent $x\simeq 0.2863$, should be compared to $\beta \mathrm{/2}\simeq 0.375$.

Finite-size scaling in networks is naturally related to the network volume *N* (number of nodes) instead of a linear size scale $\ell$, which usually cannot be defined. Assuming that $N\leftrightarrow {\ell }^{d}$, one can derive finite-size scaling functions, which are expected to hold in the conventional mean-field regime $d > {d}_{c}$, or for dense networks. This leads to a finite-size scaling function of the order parameter:

4 $${\rho }_{E}(\delta t,N)\sim {N}^{-\beta /d{\nu }^{\ast }}{\tilde{\rho }}_{E}({N}^{\mathrm{1/}d{\nu }^{\ast }}(\delta t-\delta {t}_{c})),$$

where ${\nu }^{\ast }=\mathrm{2/}d$ is the finite-size scaling exponent (of linear size), which depends on the dimension *d*. If $d < {d}_{c}$ it is the spatial correlation length exponent, and above the critical dimension *d*_*c*_ = 5 it takes the value ${\nu }^{\ast }=\mathrm{2/}{d}_{c}$^37^. At the same time a similar scaling function is expected to hold for susceptibility:

5 $${\chi }_{E}(\delta t,N)\sim {N}^{\gamma /d{\nu }^{\ast }}{\tilde{\chi }}_{E}({N}^{\mathrm{1/}d{\nu }^{\ast }}(\delta t-\delta {t}_{c})),$$

where *γ* is the mean cluster-size exponent. From the definition of *χ*_*E*_ (in Eq. (2)) and the scaling of *ρ*(*δt*,*N*) at *δt*_*c*_ we can derive the simple exponent relation $\gamma /(d{\nu }^{\ast })=1-\beta /(d{\nu }^{\ast })$, where ν* = 2/*d*, *d* = *d*_*c*_ = 4 and *β* ≃ 0.75, which gives us a value *γ* ≃ 1.25 (which is slightly different from the directed-percolation MF value of *γ*_*MF*_ = 1.0).

To check whether the predicted finite-size scaling behaviour holds around the critical point, we took the simulated *ρ*_*E*_(*δt*, *N*) and *χ*_*E*_(*δt*, *N*) measured for various *N* (see Fig. 2e and h respectively). Using the scaling functions in Eqs (4) and (5) with the determined exponents, we scaled the order parameter and susceptibility as a function of (*δt* − *δt*_*c*_). The expected scaling behaviour appears for both quantities close to the critical point (Fig. 2f and i).

### Weighted event graphs of empirical temporal networks

We next investigated temporal percolation in real-world networks. We studied three cases: (a) a mobile call network^12^ of ∼3.2 × 10^8^ time-stamped interactions over 120 days of ∼5.2 × 10^6^ of customers of an European operator; (b) a sexual-interaction network^20^ from Brazil with 16,726 sex workers and clients who interacted 42,409 times over 2,231 days; and (c) an air transportation network with the time, origin, destination and duration of 180,192 flights between 279 airports in the United States^38^ over 10 days. For details, see Materials and Methods. These networks are relevant for diffusion of information, disease, and passengers.

**Figure 3:**
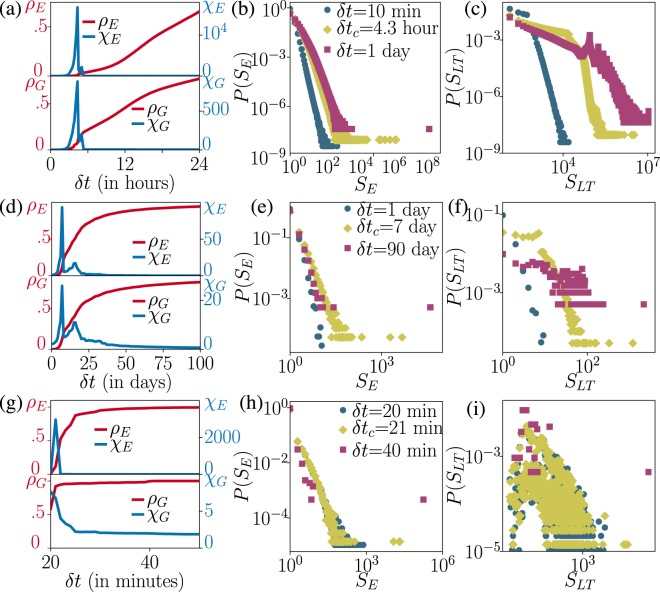
Percolation transitions in empirical temporal networks of mobile communication (**a**–**c**), sexual interactions (**c**–**e**), and air transportation (**g**–**i**). Panels (a,d,g) depict the order parameter ρ_*_(*δt*) (solid red lines) and susceptibility *χ*(*δt*) (solid blue lines) of weakly connected components with sizes in events (upper panels) and temporal-network nodes (lower panels). Panels (b,e,h) show the size distributions of weakly connected components in the event graph measured below (blue circles), at (yellow diamonds), and above (violet squares) the critical *δt*_*c*_. (**c**,**f**,**i**) are the same but depict the distribution of the lifetimes of weakly connected components in *D*.

We measured the largest weakly connected component *ρ*_*E*_ (resp. *ρ*_*G*_) and susceptibility *χ*_*E*_ (resp. *χ*_*G*_ defined similarly as Eq. (2)) in terms of events (resp. temporal-network nodes) covered by the event graph components. As seen in Fig. 3a for calls and in 3d for the sexual-interaction networks, the phase transitions of both types of components take place at similar times. The percolation point *δt*_*c*_ is identifiable as the peaks of susceptibility, with $\delta {t}_{c}\sim 4$ h 20 min for the calls and $\delta {t}_{c}\sim 7$ d for the sexual-interaction network. A spreading agent has to survive at least this long at a node to percolate the network. Interestingly, for both networks, the susceptibility shows a second peak (resp. ∼5 hours and ∼16 days), which may indicate another characteristic time-scale for the connectedness. For demonstration we computed the evolution of the largest in- and out-components for the sexual-interaction network (shown in SM), which appear with similar percolation points as the largest weakly connected component, but they evidently evolve slower in size.

For the air transportation network, we set further conditions. So far we have assumed instantaneous and bidirectional events; for flights we consider directed events with a specific duration (for details, see Materials and Methods), and require $\delta t\ge 20$ minutes for realism (achievable transfer time). As shown in Fig. 3g this network too undergoes phase transitions, first in *G* at ≈20 min, and shortly thereafter in *D*_*δt*_ at ≈21 min. At *δt* = 45 min the network becomes almost entirely connected. Note that *δt* = 21 minutes is close to a minimal transfer time while 45 min can be considered as typical.

Percolation theory suggests that the structural phase transition is reflected in the component size distributions around *δt*_*c*_. It is expected that below the critical point ($\delta t < \delta {t}_{c}$), only exponentially small components are present. At the critical point *δt*_*c*_, $P({S}_{\ast })$ appears with a power-law tail, and above *δt*_*c*_ the distribution is dominated by a single giant component while other components are exponentially small. This behaviour was found for weakly connected components with sizes measured as events or nodes for all three empirical systems (see Fig. 3b,e and h). However, for component lifetimes, there can be many giant components: while for small *δt*, the tail of *P*(*S*_*LT*_) appears as a power-law, for larger *δt* long-lived components may coexist that span the observation period but involve only a vanishing fraction of network nodes and events.

The weakly connected components discussed above only provide an upper bound for the largest reachable set of a spreading process. For a more precise measure, one to obtain the largest *out*-*component* which is computationally costly. This sets an important future direction: to develop an algorithmic solution for identifying the largest out-component of any node in a dense, directed, and large acyclic graph.

## Discussion

To summarize, we have introduced a new representation of temporal networks by mapping them into event graphs that are static, weighted, directed, and acyclic. Weighted event graphs recast temporal networks as static graphs that encode their topological and temporal structure without loss of information, greatly reducing the computational cost of temporal-network studies. They make it possible to use the methodology developed for directed acyclic graphs–and for time-invariant structures in general–in the analysis of temporal networks. This representation contains all time-respecting paths of a temporal network and easily yields their *δt*-constrained subset. Temporal paths are important as they determine if the diffusion of any kind of information between nodes is possible. Therefore they are pivotal for identifying the percolating structure of temporal networks as well as the outcome of any dynamical process unfolding on them. There are many examples from epidemic processes transmitted via social interactions to the routing of passengers in a transportation systems, where the timing of travel services defines the underlying temporal structure. In addition to percolation studies, weighted event graphs can be used to compute centrality scores for events, links, and nodes, and to quickly identify the complete set of *δt*-connected temporal motifs^39^. They can be used to study diffusion-like processes without having to compute average outcomes of stochastic simulations. Further they open new directions for studying system-level higher-order correlations in temporal networks.

## Materials and Methods

### Temporal networks with durations/delays on events

In some temporal systems it is not enough to consider the times of events, but one needs to also consider the durations (or delays) of the events^3^. In this case we define a set of events as $E\subset V\times V\times \mathrm{[0},T]\times [0,T]$, where in an event (*u*, *v*, *t*, *t*_*d*_) ∈ *E* the additional member *t*_*d*_ represents the duration or delay related to the event. This additional element is necessary for example in the air-traffic network studied here, where the *t*_*d*_ represents the flight times, and allows us to consider *δt*-adjacent time-respecting paths that can correspond to actual trips taken in the system.

The *δt*-adjacency in systems with duration or delay are defined exactly as in the simpler systems but with the allowed time difference between two events *e* = (*u*, *v*, *t*, *t*_*d*_) and $e^{\prime} =(u^{\prime} ,v^{\prime} ,t^{\prime} ,{t^{\prime} }_{d})$ being defined as $0 < t^{\prime} -t-{t}_{d} < \delta t$. Note that this is not a separate definition for the *δt*-adjacency but a generalisation of the case without durations, as the *δt*-adjacency defined in the main text is returned when all events have *t*_*d*_ = 0.

### Definitions of *δt* adjacencies for directed networks

In directed networks spreading, diffusion, and progress of other dynamics are constrained by the direction of the edges in addition to the arrow of time. This can be taken into account in the *δt*-adjacencies by restricting the adjacencies where *e* → *e′* only when in the two events *e* = (*u*, *v*, *t*, *t*_*d*_) and $e^{\prime} =(u^{\prime} ,v^{\prime} ,t^{\prime} ,{t^{\prime} }_{d})$ have $v=u^{\prime}$. The air-traffic network studied in the main text is considered directed in this way.

### Algorithm to construct weighted event graph representation of temporal networks

Constructing the weighted event graph representation of a temporal network *D* = (*E*, *E*_*D*_, *w*) can be done efficiently by noting that the edges in *E*_*D*_ can be listed by inspecting the sequence of events around each node *v* ∈ *V* separately. For some data sets the full weighted event graph *D* might be large, and it is convenient to construct ${D}_{\delta {t}_{{\rm{\max }}}}$ that can, for example, be later used to sweep through all values $\delta t < \delta {t}_{{\rm{\max }}}$.

For each node in the temporal network *v* ∈ *V* one can build a time-ordered sequence of events {*e*_1_, …, *e*_*k*_} in which *v* participates. In the case where there are no durations one can then simply iterate over each event *e*_*i*_, and for each of them search forward in the ordered event sequence until one finds an event *e*_*j*_ for which ${t}_{j}-{t}_{i} > \delta {t}_{{\rm{\max }}}$. One then adds a link *e*_*i*_ → *e*_*j*_ at the each step of this process until the event *e*_*j*_ that is too far from the starting event *e*_*i*_ is found. (Note that some *δt* adjacencies are found twice.) Creating the event sequences and sorting them can be done in ${\mathscr{O}}(|E|\,\mathrm{log}\,|E|)$ time, and as each step of the algorithm produces a single link (with possibility of some links being visited twice) the algorithm runs in total ${\mathscr{O}}(|E|\,\mathrm{log}\,|E|+|{E}_{D}|)$ time. Including the durations of events only requires a small adjustment to this algorithm, for example, a construction of sequences of events that are sorted according to the end times of the events *t* + *t*_*d*_.

1:  **function** Weighted event graph edges for a node{*e*_1_, …, *e*_*k*_}

2:        **for** $$i\leftarrow 1\,{\rm{to}}\,k$$ **do**

3:               *j* ← *i* + 1

4:               **while** ${t}_{j}-{t}_{i}\le \delta {t}_{{\rm{\max }}}$ and $j\le k$**do**

5:                    Output: *e*_*i*_ → *e*_*j*_

6:                    *j* ← *j* + 1

### Extracting component distributions from a weighted event graph

The weighted event graph representation can turn problems related to dynamics of temporal networks to problems of graph structure, and this allows one to take advantage of computational methods developed for analysing massive graphs.

The *δt* adjacencies within a specific range $\delta t < \delta {t}_{{\rm{\max }}}$ can be calculated via “thresholding” the full network (i.e., removing all edges above the threshold level $\delta {t}_{{\rm{\max }}}$). In static weighed networks the weakly-connected component distributions can be calculated for all possible threshold levels very efficiently by threshold sweep, where edges are added to the network in ascending order of their weights. This typical approach in network percolation studies^40^ can be even completed without explicitly constructing the network but by only updating a disjoint-sets forest data structure^41^. As the weighted event graph representation is a static weighted graph, these procedures can be used for finding the component size distribution in terms of events *S*_*E*_ (and quantities derived from it) for very large temporal networks and for a range of *δt* values. In practice the limit is the number of events that can be stored in memory. With some modifications, the standard algorithms can also be used to find the component size distributions in terms of the number of temporal network nodes *S*_*G*_ and component life times *S*_*LT*_.

For problems related to reachability in the weighted event graph, which is a directed acyclic graph (DAG), one can simplify the full network of all *δt* connections by removing loops via transitive reduction. Removing all loops in large networks is an expensive procedure, but removing local loops, for example, around a single node in the network construction process described above is fast. Note that in the special case, when networks are undirected and no durations are present, this local procedure gives the same result as the one described recently and independently in ref.^32^.

There are several other ways of making computing various quantities faster that are apparent when the temporal network is represented as a weighted event graph. Any algorithms developed for DAGs, for example, to calculate reachability or transitive closures, are immediately useful in the context of temporal networks. Further, new algorithms can be easily developed with specific temporal network problems in mind. For instance, consider a case where one is interested in finding the maximum size of the out-component, which would guarantee that any process following *δt*-connectivity could not affect more than that many events in the temporal network. A naïve solution, that could be devised even without considering temporal event graphs, would be to start a breadth first search from each event in the temporal network and find the maximum. However, in the temporal event graph representation it is apparent that one can omit this search for nodes which have non-zero in-degree, and also for those belonging to weakly-connected components which are smaller than the maximum size found in previous searches.

### Detailed data description

We utilised three datasets in this study, each recording a temporal network as a sequence of events. They were:

- Mobile call interaction network^12^ recording 324,528,907 millions of temporal interactions over 120 days of 5,193,086 millions of customers of a single provider in an undisclosed European country.
- Sexual-interaction network^20^ recorded in Brazil with the involvement of 16,726 sex workers and clients who interacted 42,409 times over 2,231 days.
- Air-transportation network containing the time, origin, destination and duration of 180,192 flights between 279 airports in the United States^38^ over 10.3 days.

Details of each dataset are also summarised in Table 1. We chose these three empirical networks as they record rather different types of interactions (resp. communication, social, and transportation) and in turn are important to disseminate different types of dynamical processes (resp. the diffusion of information, epidemics, and passengers).

**Table 1:** Summary of details about the utilised datasets.

Dataset	|*E*|	|*V*|	*T*	Resolution
Mobile calls	324,528,907	5,193,086	120 days	1 sec
Sexual interactions	42,409	16,726	2,231 days	1 day
Air transportation	56,112	279	10 days 3 hours	1 min

In data two events with no duration (i.e., zero duration) can occur in exactly the same time due to limitation in temporal resolution or due to other reason. This type of events are rare in our data sets, but they can induce loops in the network of events. In order to retain the acyclic property of these graphs we kept only a randomly selected event in case of simultaneous events of the same node.

### Data availability statement

The sexual contact network data and public transportation data we used in Fig. 3 is openly available online^20,38^. The mobile call data used in Fig. 3 is shared by an undisclosed mobile operator with restrictions on availability of these data. The data was used under license for the current study, and so are not publicly available. Data are however may be available from the authors upon reasonable request and with permission of the provider.

## Electronic supplementary material

Supplementary Materials
