# Supplementary material for: Mapping temporal-network percolation to weighted, static event graphs
Source: Sci Rep. 2018 Aug 17;8:12357. doi: 10.1038/s41598-018-29577-2 (PMC6098025; doi:10.1038/s41598-018-29577-2)
Supplement: Supplementary file 1 — Supplementary Materials [file 41598_2018_29577_MOESM1_ESM.pdf]

Supplementary Materials

Mapping temporal-network percolation to weighted, static event graphs

M. Kivelä, J. Cambe, J. Saramäki and M. Karsai

1 Event graph percolation in sexual-contact networks

As we discussed in the main text, we conducted our percolation analysis using weakly connected components of the δt thresholded weighted event graph. Computation of weakly connected components is feasible but these components provide only an upper bound for in- and out-components capturing precisely the influencing and influenced set of events of each events. To demonstrate how tight this bound is, we computed the largest in- and out component in case of the sexual-contact network. As shown in Fig. 1, although these components evolve evidently slower as compared to the largest weakly connected component, the percolation point of the three components seem to be matching. This demonstrates that, by calculating the largest weakly connected component, we overestimate the order parameter of a real spreading process, however the critical behaviour is similar for the three component definitions.

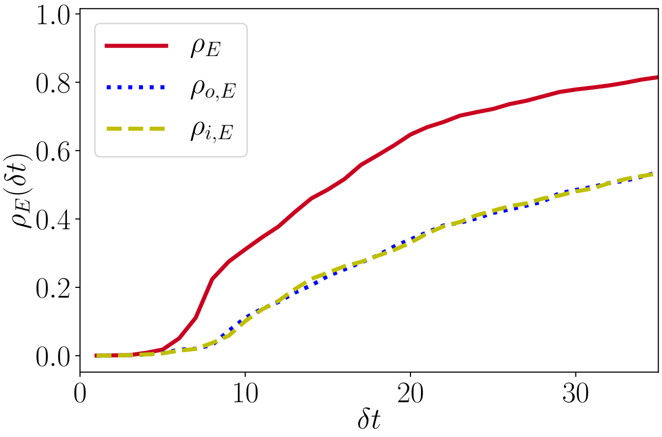

Figure 1: The scaling of the largest weakly- (red solid line), out- (blue dotted line), and in- (yellow dashed line) components, $\rho_E(\delta t)$, of the event graph as the function of δt for the sexual-interaction network.
